# Hospital Officials' Pensions

**Published:** 1922-05

**Authors:** 


### Hospital Officials' Pensions.

*To the Editor of* THE HOSPITAL & HEALTH REVIEW.

SIR,—The employees in the secretariat of the Voluntary Hospitals are indebted to you for the admirable article which appeared in your March issue. That Hospital Boards recognise, apart from any established system, the claim to pension of their employees is evident from the accounts shown in every annual report, where items appear under that heading. But there are at times striking inequalities in the treatment of retiring officers. A case lately occurred in one of the large hospitals in London where an officer of 35 years' service was pensioned at a rate far in excess of that provided by either the Civil Service Superannuation Acts or the more recent Federated Superannuation System for the staffs of Universities. It might just as well have happened, as it not infrequently has done, that this employee had been pensioned at a rate less instead of more favourable than the systems mentioned. Surely it would be better for the hospitals to face the position and adopt forthwith a general scheme of pensions, thus enabling their officers to enjoy, in the same way as almost everyone else in an official and graded position, the assurance of reasonable superannuation.

I am, &c.,
HOSPITAL SECRETARY.

